# Potential Antidepressant Effects of Scutellaria baicalensis, Hericium erinaceus and Rhodiola rosea

**DOI:** 10.3390/antiox9030234

**Published:** 2020-03-12

**Authors:** Fiona Limanaqi, Francesca Biagioni, Carla Letizia Busceti, Maico Polzella, Cinzia Fabrizi, Francesco Fornai

**Affiliations:** 1 Department of Translational Research and New Technologies in Medicine and Surgery, University of Pisa, Via Roma 55, 56126, Pisa, Italy; f.limanaqi@studenti.unipi.it; 2 I.R.C.C.S. Neuromed Pozzilli, Via Atinense, 18, 86077, Pozzilli, Italy; francesca.biagioni@neuromed.it (F.B.); carla.busceti@neuromed.it (C.L.B.); 3 Aliveda Laboratories, Viale Karol Wojtyla, 19, 56042 Lorenzana, (PI), Italy; maico@aliveda.com; 4 Department of Anatomy, Histology, Forensic Medicine and Orthopedics, Sapienza University of Rome, Via A. Borelli 50, 00161, Rome, Italy; cinzia.fabrizi@uniroma1.it

**Keywords:** phytochemicals, depression, anxiety, monoamines, neuroprotection, neurogenesis, neurotrophic factors, antioxidant, anti-inflammatory

## Abstract

Recent studies focused on the pharmacology and feasibility of herbal compounds as a potential strategy to target a variety of human diseases ranging from metabolic to brain disorders. Accordingly, bioactive ingredients which are found within a variety of herbal compounds are reported to produce both neuroprotective and psychotropic activities which may help to combat mental disorders such as depression, anxiety, sleep disturbances and cognitive alterations. In the present manuscript, we focus on three herbs which appear effective in mitigating anxiety or depression with favourable risk-benefit profiles, namely Scutellaria baicalensis (*S. baicalensis*), Hericium erinaceus (*H. erinaceus*) and Rhodiola rosea (*R. rosea*). These three traditional folk medicinal herbs target the main biochemical events that are implicated in mental disorders, mimicking, to some extent, the mechanisms of action of conventional antidepressants and mood stabilizers with a wide margin of tolerability. In detail, they rescue alterations in neurotransmitter and neuro-endocrine systems, stimulate neurogenesis and the synthesis of neurotrophic factors, and they counteract oxidative stress, mitochondrial dysfunction and inflammation. Albeit the encouraging results that emerge from both experimental and clinical evidence, further studies are needed to confirm and better understand the mental-health promoting, and specifically, the antidepressant effects of these herbs.

## 1. Introduction

A growing number of people worldwide suffer from age- or stress-related mental disorders such as depression, anxiety and insomnia. Since many conventional medications possess either side effects or limited efficacy, many patients increasingly prefer herbal compounds for such mood symptoms relief [1]. Accordingly, recent investigations have focused on the psychopharmacology of naturally-occurring compounds as a potential strategy to target mental disorders. Wide evidence indicates that bioactive ingredients found within a variety of phytochemical compounds are endowed with multiple, yet intricate, psychotropic activities which may help to combat depression, anxiety, sleep disorders and cognitive alterations [2]. Several phytochemicals are reported to produce mental benefits that are comparable to standard anxiolytics and antidepressants in the absence of overt adverse effects [1,2]. As such, they appear as an optimal, first-choice therapy in mild-to-moderate depressive disorders. Furthermore, some herbal extracts may be also combined or enriched with conventional antidepressants and mood stabilizers to alleviate some of their common side effects while potentiating their efficacy [3,4]. 

In the present manuscript we focus on three neuroprotective herbs which appear effective in mitigating anxiety or depression with favourable risk-benefit profiles, namely Scutellaria baicalensis (*S. baicalensis*) [5,6,7,8,9], Hericium erinaceus (*H. erinaceus*) [10,11,12,13,14] and Rhodiola rosea (*R. rosea*) [15,16,17,18,19,20]. We chose these specific herbs in order to analyse both their overlapping and complementary properties, posing the provocative issue of whether their combined administration may yield synergistic antidepressant-like effects. The general health-promoting effects of *S. baicalensis, H. erinaceus* and *R. rosea* in humans and animals are mainly attributable to anti-inflammatory and antioxidant properties [6,7,8,16,17,18,19,21,22,23,24,25,26,27,28,29,30,31]. 

**Figure 1:**
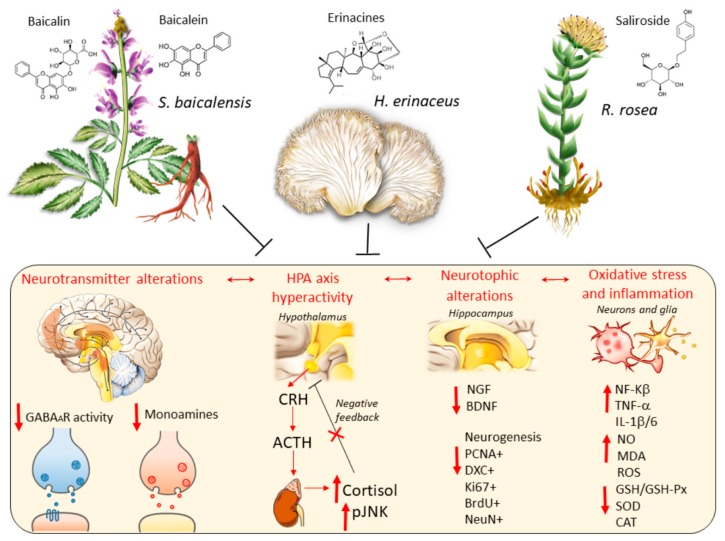
A general view on the mechanisms of action of *S. baicalensis*, *H. herinaceum* and *R. rosea* against the main biological pathways that are altered in depression and depressive-like behavior (yellowish box). Hypotheses on the neurobiology of depression and anxiety are largely based on dysregulations of (i) neurotransmitter systems, including GABA and GABA_A_ receptors [8], as well as monoamines (dopamine, norepinephrine and serotonin) [9]; (ii) hypothalamic-pituitary-adrenal (HPA) axis consisting of an abnormal release of corticotropin-releasing factor (CRH), adrenocorticotropin (ACTH) secretion from the anterior pituitary, abnormal secretion of glucocorticoids (cortisol in humans and corticosterone in rodents), activation of phosphorylated c-Jun kinase (pJNK), and aberrant negative feedback HPA axis [9]. (iii) impaired neurotrophic mechanisms consisting of low expression of nerve growth factor (NGF) and brain-derived neurotrophic factor (BDNF), impaired neurogenesis and plasticity [9]; (iv) chronic oxidative stress and neuroinflammation [10]. Black T-shaped lines indicate inhibition. Red bold arrows indicate downregulation or hyperactivation. Red thin arrows indicate sequential molecular events.

At a first glance, the mechanisms of action of these herbal compounds in mental diseases appear puzzling and often overlapping. The bioactive ingredients found within *S. baicalensis, H. erinaceus* and *R. rosea* target the main biochemical events which are implicated in psychiatric conditions [32,33,34] mimicking at some extent the mechanisms of action of conventional antidepressants and mood stabilizers in the absence of serious adverse effects [5,10,13,15,20,35,36] (Figure 1). Far from being the aim of the present manuscript is an attempt to deal with the neurobiology of depression, which is a complex, multifactorial disorder [33], here we limit to reviewing and discussing potential biochemical and molecular mechanisms through which the abovementioned compounds produce anxiolytic/antidepressant-like effects. In detail, bioactive compounds within *S. baicalensis, H. erinaceus* and *R. rosea* rescue alterations in monoamine and GABA neurotransmission, and they stimulate neurogenesis and the synthesis of neurotrophic factors while counteracting oxidative stress, mitochondrial dysfunction and inflammation (Figure 1) [5,6,7,8,9,10,11,12,13,14,15,20]. These herbs also modulate neuro-immune and neuro-endocrine functions by targeting hypothalamic-pituitary-adrenal (HPA) axis hyper-activation, which is implicated in mental disorders (Figure 1) [1,19,33]. Nonetheless, at a closer examination, each plant possesses specific effects by acting on different neurotransmitter systems and molecular pathways. After briefly summarizing the main herbal-related neuroprotective mechanisms which may be relevant for depressive disorders, we move to discuss evidence centred on the anxiolytic/antidepressant potential of each herb, as assessed specifically in experimental and clinical studies. 

The still limited, although encouraging, results which emerge from both experimental and clinical studies underline the need for further investigations aimed at dissecting the fine molecular mechanisms of action as well as the safety and pharmacokinetic profiles of these herbal compounds. 

## 2. *S. baicalensis*, *H. erinaceus* and *R. rosea*: Toxicology and Neuroprotective Effects which may be Relevant for their Antidepressant Potential

*S. baicalensis*, *H. erinaceus* and *R. rosea* are folk traditional medicinal herbs that gained increasing popularity for their health-promoting properties including antitumor, hepatoprotective, antimicrobial, anti-inflammatory, anti-hyperlipidemic, antidiabetic, cardio-protective, neurotrophic and neuroprotective effects [5,10,15,16,35,37,38,39,40,41,42,43,44,45,46,47]. In the brain, the beneficial effects of each herb are due to different bioactive compounds, some of which are able to cross the blood-brain-barrier (BBB). For *S. baicalensis* these correspond to two major flavonoids, namely baicalin (glucuronide) and baicalein (aglycon), being purified from the plant’s dry roots (*Scutellariae* radix) [48]. *H. erinaceus* possesses three main classes of bioactive compounds, namely polysaccharides, hericenones and erinacines, with the first two being extracted from the fruit bodies and the latter from the mycelia. Despite the widely reported beneficial effects of these *H. erinaceus* bioactive ingredients in the brain, to date only erinacines have been documented to cross the BBB [49]. Eventually, the main bioactive compound of *R. rosea* is salidroside glycone, which has been detected in the brain tissue as well [29,50,51]. 

Based on toxicological and clinical studies, *S. baicalensis*, *H. erinaceus* and *R. rosea* are generally considered to be safe and well tolerated [40,52,53,54,55,56,57,58,59,60,61,62,63]. In experimental studies, the estimated median lethal dose (LD_50_) of some compounds varies according to the extraction method and administration route [54]. In fact, as reported by pioneer toxicological studies, following subcutaneous injection in mice, the median lethal dose (LD_50_) of both *S. baicalensis* ethanolic extract and isolated baicalin is 6 g/kg [54]. Instead, the LD_50_ of isolated baicalin following intraperitoneal injection is 3.081 g/kg [54]. More recently, 2.5 g/kg of *S. baicalensis* ethanol extracts were shown to be safe in rats, though some reversible inflammatory changes were detected in the liver [55]. Baicalin was shown to inhibit the proliferation of embryonic stem cells at half maximal inhibitory concentration (IC_50_) values up to 135.9 mg/l, suggesting that it may induce low embryonic toxicity at high concentrations [56]. In humans, oral intake of *S. baicalensis* extracts and baicalin at the daily doses of 300 mg and 200–800 mg respectively, is generally safe and well tolerated [57,58]. 

As far as it concerns *H. erinaceus,* the acute oral LD_50_ of its erinacine-A-enriched mycelia is higher than 5 g/kg in rats [59]. As shown by sub-chronic toxicology studies, erinacine-A-enriched *H. erinaceus* administered daily for 28 days or 13 weeks is safe and not teratogenic at doses up to 3 g/kg and 2.625 g/kg, respectively, which is nearly 171 times the recommended daily intake for humans (1.05 g/60 kg of body weight/day) [60,61]. Moreover, *H. erinaceus* mycelium is not mutagenic in the bacterial reverse mutation test (Ames test), *in vitro* chromosome aberration test, and *in vivo* erythrocyte micronucleus test [61]. In line with these studies, in rats, no toxicity signs are observed following oral administration of an *H. erinaceus* mycelia extract at 2.395 g/kg [49]. In humans, no adverse effects are reported following oral intake of *H. erinaceus* extracts at the cumulative dose of 1.650 g/day (of which 80% bulk mycelia, and 20% fruiting body extracts) [62]. 

Pioneer acute toxicity studies showed that *R. rosea* possesses low toxicity in rats, with LD_50_ being estimated as 3.360 g/kg [63]. However, in more recent toxicological studies on *R. rosea*-treated mice and A. salina brine shrimp, neither the LD_50_ >5.000 g/kg nor the LC_50_ >1.000 mg/ml exhibited toxic effects [64]. Salidroside, the main active component of *R. rosea* is not genotoxic at doses up to 1.5 g/kg in mice and it does not lead to maternal or embryonic toxicity at 0.125 g/kg in rats [65,66]. In summary, based on known toxicological data on animal studies, *R. rosea is* generally evidenced to be safe, with no acute and chronic toxicity under the experimental conditions at its therapeutic window [53,64]. In line with this, oral intake of *R. rosea* at does up to 680 mg/day does not produce any serious adverse effects in humans [67]. 

### 2.1. Common Neuroprotective Effects of S. baicalensis, H. erinaceus and R. rosea 

**Figure 2:**
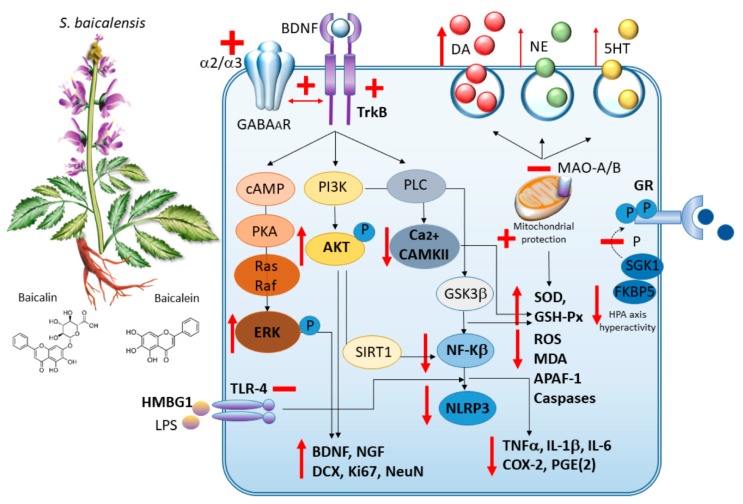
*S. baicalensis*: molecular mechanisms underlying neuroprotection and anxiolytic/antidepressant-like effects. By acting as MAO A/B inhibitors, *S. baicalensis* and its bioactive ingredients (mostly baicalin and baicalein) induce monoamine, and mostly DA release. At the same time, they act as partial, subtype-selective GABA_A_ receptor ligands and also foster the interaction of GABA_A_ receptors with TrkB to potentiate GABA-induced signaling. By increasing cAMP/pERK and PI3K/pAKT signaling, they promote the synthesis of neurotrophic factors (BDNF and NGF) as well as neurogenesis. *S. baicalensis* promotes anti-inflammatory effects through inhibition of HMBG1/TLR4/NF-k, AKT/SIRT1/NF-k, and GSK3/NF-k axes. This leads to a reduction in NLRP3 and proinflammatory cytokines levels. At the same time, *S. baicalensis* promotes anti-oxidant effects which are bound to both Ca2+/CAMKII pathway inhibition and mitochondrial protection. By downregulating SGK1 and FKBP5, *S. baicalensis* also counteracts alterations in glucocorticoid receptors (GR) associated with phosphorylation of its crucial serine residues and HPA axis hyperactivity.

*S. baicalensis*, *H. erinaceus* and *R. rosea* produce neuroprotective effects in models of Parkinson’s disease (PD) [6,7,12,17,68,69], Alzheimer’s disease (AD) [11,70,71,72,73,74,75,76,77,78,79], Huntington disease (HD) [18], hypoxia/hypo-perfusion/stroke [51,80,81], nerve and brain injury [82,83], glutamate-induced neurotoxicity [16,75,84], and epilepsy [85,86,87]. The in vitro and in vivo neuroprotective effects of these herbal compounds, administered as either full/enriched herbal extracts and/or as single bioactive ingredients, are largely attributable to antioxidant, mitochondrial-protecting and anti-inflammatory activities (Figure 2). 

**Figure 3:**
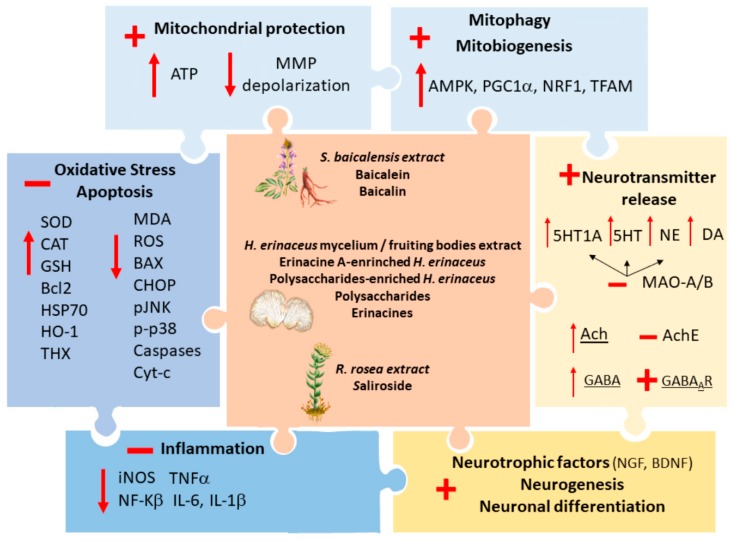
Mechanisms underlying the neuroprotective effects of *S. baicalensis, H. erinaceus and R. rosea*. The neuroprotective effects of *S. baicalensis, H. erinaceus and R. rosea*, administered as either full/enriched herbal extracts and/or as single bioactive ingredients (central box), are bound to antioxidant, mitochondrial-protecting, anti-inflammatory, neurotransmitter release-stimulating and neurotrophic/neurogenic activities. These effects are here represented as pieces of a puzzle where *S. baicalensis, H. erinaceus and R. rosea* perfectly fit in the middle. Antioxidant properties of these compounds consist of reducing the levels of reactive oxygen species (ROS), enhancing superoxide dismutase (SOD), glutathione (GSH) and catalase (CAT) activities along with heat shock proteins 70 (HSP70), heme oxygenase-1 (HO-1) and thioredoxin (THX) levels, while decreasing lipid peroxidation (malondialdehyde, MDA, content) and counteracting apoptosis through reduction of Bax, C/EBP Homologous Protein (CHOP), pJNK, pp38, caspases 3, 6 and 9, and cytochrome-c release. These effects are closely associated to mitochondrial protection, namely improvement of mitochondrial membrane potential (MMP) depolarization and ATP production, and concomitant enhancement of mitophagy and mitochondrial biogenesis via increasing 5’ AMP-activated protein kinase (AMPK), Peroxisome proliferator-activated receptor gamma coactivator 1-alpha (PGC1α), Nuclear respiratory factor 1 (NRF1) and Mitochondrial transcription factor A (TFAM). At the same time the three herbs promote anti-inflammatory effects by decreasing NF-kβ and iNOS enzymes levels while inhibiting the release of pro-inflammatory cytokines TNF-α, IL-6 and IL-1β. These herbs also target alterations of neurotransmitter systems, by enhancing the release of monoamine through MAO inhibition, acetylcholine (Ach) via inhibition of acetylcholine esterase (AchE), and GABA-induced inhibitory currents by promoting stimulation of GABA_A_ receptors.

In detail, common antioxidant properties of these compounds consist of reducing the levels of reactive oxygen and nitrogen species (ROS and RNS, respectively), enhancing superoxide dismutase (SOD), glutathione (GSH), glutathione peroxidase (GSH-Px) and catalase (CAT) activities along with heat shock proteins 70 (HSP70), heme oxygenase-1 (HO-1) and thioredoxin levels, and eventually, decreasing lipid peroxidation assessed as reduction of malondialdehyde (MDA) content and lipoxygenase (LPX) inhibition [6,7,12,17,26,27,28,69,70,73,75,78,79,81,85,86,87]. They also counteract mitochondrial alterations, endoplasmic reticulum (ER) stress, and apoptosis by improving mitochondrial membrane potential (MMP) depolarization and ATP production while promoting mitophagy and mitochondrial biogenesis, and by decreasing the levels of C/EBP Homologous Protein (CHOP), pJNK, p-p38, Bax/Bcl-2 ratio, caspases 3, 6 and 9, and cytochrome-c release [12,16,51,68,69,71,73,75,77,78,81,83]. Again, *S. baicalensis*, *H. erinaceus* and *R. rosea* counteract inflammation by decreasing glial cells activation, inducible nitric oxide synthase (iNOS) and NF- κB levels, as well as the production of pro-inflammatory cytokines TNF-α and IL-β1 and IL-6 [8,16,29,74,79,83] (Figure 3). Despite being assessed in neurotoxicity models, these effects may be also key for depressive-related disorders, where oxidative stress, mitochondrial alterations and inflammation are widely implicated [34]. As a further common effect which is relevant to both neurodegenerative and mental disorders, these herbs also promote neurogenesis, neuronal differentiation and the synthesis of neurotrophic factors [8,12,72,74], and they enhance the release of dopamine (DA), norepinephrine (NE) and serotonin (5-HT), in part by acting as monoamine oxidase (MAO) inhibitors [6,7,12,88,89,90,91]. Nonetheless, at a closer look, each herb also possesses specific effects which may complementarily contribute to their mood-stabilizing potential beyond neuroprotection. These effects are discussed in the following section before moving to experimental and clinical studies on depression. 

### 2.2. Complementary Effects of S. baicalensis, H. erinaceus and R. rosea

*S. baicalensis* primarily targets alterations of the DA system, which are implicated in various neurological and mental disorders, and also in their comorbidity [92]. Immediately after intravenous administration, total flavonoids from *Scutellariae* radix are able to cross the BBB, with the striatum and hippocampus being the most prominent targets [88]. Remarkably, baicalin distributes most specifically to the DA system and it induces an increase in DA levels in the rat striatum, hippocampus and cortex [88,89,93]. Accordingly, recent studies suggest that baicalin and baicalein may induce beneficial effects in DA-related brain disorders by increasing DA levels in the brain besides protecting dopaminergic neurons from mitochondrial- and oxidative-related toxicity [6,7,94]. In fact, baicalein fully prevents 6-OHDA- and MPTP-induced behavioral alterations by preventing reductions of striatal DA levels, the increase in DOPAC/DA and HVA/DA ratios and the loss of striatal tyrosine hydroxylase (TH) [6,7]. Similarly, baicalin prevents methamphetamine (METH)-induced alterations, namely the loss of DA and DA transporter (DAT) in the striatum, which play an important role in the pathogenesis of mental disorders [94]. Baicalin also ameliorates synaptogenesis and memory-related dysfunctions which are associated with GABA_A_ receptor downregulation following abnormal stimulation of DA D1 receptors (D1Rs) [95]. In detail, baicalin prevents the reduction of GABA_A_-induced currents which occurs following the *in vivo* administration of exogenous DA and abnormal stimulation of D1Rs [95]. Mechanistically, baicalin fosters the interaction of GABA_A_R with tyrosine kinase receptor B (TrkB) and AKT thus reversing the DA-induced decrease in the expression of GABA_A_R/TrkB/AKT pathway. Thus, baicalin plays a key role in modulating the GABAergic system. This is supported by the emerging role of baicalin as a potential anxiolytic by acting as a partial, subtype-selective GABA_A_ receptor ligand [9]. These findings are in line with increasing evidence centered on the neuroprotective and nootropic action of *Scutellariae* radix extracts in substance-induced addiction, attention deficit hyperactivity disorders, depression and anxiety [93,95,96]. 

*H. erinaceus* has a prominent effect on neurotrophic factors’ induction and modulation of NE system. In detail, *H. erinaceus* bioactive compounds, especially erinacines and hericones, possesses strong nerve growth factor (NGF)-stimulating properties, showing remarkable neurite outgrowth activities in various cell lines and in dissociated cells of brain, spinal cord, and retina [40,97,98,99,100,101,102,103,104,105,106,107,108]. In detail, *H. erinaceus* enhances NGF-mediated neurite outgrowth via activation of the Trk/MEK/ERK and PI3K-Akt signalling pathways [105,106]. Recently, two novel *H. erinaceus* cyathane diterpenoid derivatives were shown to promote BDNF expression *in vitro*, suggesting a common, yet unknown, upstream target for both NGF and BDNF induction [109]. Intriguingly, in vivo administration of erinacine A-enriched *H. erinaceus* extract produces an increase in NGF levels which is detected specifically within the major NE-producing brainstem nucleus Locus Coeruleus (LC) and within the hippocampus of rats [90]. Remarkably, such an effect matches the increase in *H. erinaceus*-induced NE levels within the LC and hippocampus, suggesting that *H. erinaceus,* and mostly erinacines, may modulate neurotrophin-neurotransmitter interactions, especially in the LC-hippocampal axis. This is key since NE-LC neurons are critically involved in stress-response, depression and sleep disturbances; in fact, different stress-related molecular changes related to the LC have been detected in patients with mood disorders, and LC-CA1 hippocampal projections are decreased in mice models of chronic social defeat stress (CSDS) and chronic footshock stress (CFS) [110,111]. Remarkably, an early degeneration of NE-LC neurons occurs in AD [112], and such a phenomenon was recently linked to depressive symptoms characterizing early AD stages [113]. In detail, a minimal loss of NE-LC neuronal population following a bilateral infusion of the neurotoxin 6-OHDA produces depressive-like behaviors in mice, which can be reversed by administration of NE precursors [113]. Thus, by stimulating NE and NGF/BDNF synthesis, *H. erinaceus* may produce plastic effects which are expected to counteract behavioural alterations besides neurotoxicity. In fact, *H. erinaceus* reverses early learning and memory deficits which are induced by amyloid beta peptides independently of neuropathology in AD mice models [114]. 

Eventually*, R. rosea* possesses remarkable multi-target activities on both cellular and systemic levels of stress-response regulation [115]. As assessed through RNA microarray studies in neuroglial cell lines, *R. rosea* modulates the transcription of various biological and molecular mediators that are associated with emotional behavior, particularly aggressive behaviour [116]. In detail, *R. rosea* regulates various components of the antioxidant, anti-inflammatory, neuroendocrine, neurotrophic and neurotransmitter receptor pathways, which are likely associated with both its neuroprotective potential and beneficial effects on mood [15]. *R. rosea* increases the levels of both monoamines and acetylcholine (Ach) in nerve terminals and is likely bound to its mental health-promoting effects concerning both mood and cognition [91,115,116,117,118,119,120,121]. However, compared with *S. baicalenis* and *H.* erinaceus, *R. rosea* produces more significant effects at the level of the 5-HT system, by increasing 5-HT and 5-HT1A receptor levels [121,122]. *R. rosea* also produces remarkable anti-stress effects by modulating HPA axis and opioid peptides release and by acting as a corticotrophin-releasing factor (CRF) antagonist, thus blunting cortisol release [37,115,118]. Again, *R. rosea* inhibits stress-induced molecular events including abnormal cortisone release, nitric oxide production and pJNK expression [123]. At the molecular levels, *R. rosea* inhibits stress-induced over-activation of the pSAPK/pJNK pathway, which is bound to oxidative stress as well as altered synaptic plasticity and glucocorticoid receptors (GR) responsivity, and this is specifically implicated in its antidepressant-like effects [36,123]. At the same time, *R. rosea* stimulates the release of stress-related molecules, namely neuropeptide Y (NPY) and Hsp72, which likely represents a defense cellular response aimed at increasing tolerance and adaptation to stress [124]. These findings are in line with a growing body of both preclinical and clinical evidence indicating its potential use in the prevention and treatment of stress- and age-related cognitive and mood alterations, including fatigue, weakness, depression and anxiety [15,43,110,115,117,125,126,127]. 

## 3. *S. baicalensis*, *H. erinaceus* and *R. rosea*: Anxiolytic/Antidepressant Effects in Experimental and Clinical Studies

### 3.1. S. baicalensis in Experimental Models

#### 3.1.1. Anxiety 

The anxiolytic-like effects of *S. baicalensis* have been demonstrated since the early 1990s [128]. Administration of *S. baicalensis* extract and baicalin was shown to normalize all of the hormonal metabolic disturbances which develop in rats exposed to stress by fixation. These include alterations in insulin, urea, glucose, corticotrophin and hydroxycorticosteroids levels, which are bound to alterations of hypothalamic and adrenocortical functional activity [128].

Recently, baicalin emerged as an alternative, GABA_A_ receptor benzodiazepine (BZ)-site ligand possessing minimal side effects along with a selective activity profile compared with BZs [9,129,130,131]. Despite common prescription of conventional BZs as potent anxiolytics, the numerous side effects they produce such as sedation, myorelaxation, amnesia, and addiction, have continuously prompted the search for alternative BZ site ligands with less side effects. Baicalin was identified as a BZ-site ligand since the early 2000s [129], and this was associated with anxiolytic-like activity occurring in the absence of sedative and myorelaxant side effects, as assessed in mice through Vogel conflict test and elevated plus maze test [130,131]. 

Subsequent studies also assessed the effects of baicalin on GABA_A_ receptor binding selectivity as well as on the cognitive impairment, anticonvulsant and motor incoordination side effects that are associated with conventional anxiolytics [9]. Contrarily to diazepam (3 mg/kg), baicalin (3.3–30 mg/kg) acts as a subtype-selective partial agonist of GABA_A_ receptors, exhibiting no amnesic, anticonvulsant, and motor incoordination activities in mice, as assessed through the step-through passive avoidance test, picrotoxin-induced seizure test and rotarod test, respectively. Contrarily to the full agonist diazepam, baicalin shows a selectivity profile by acting through α2- and α3-containing GABA_A_ receptor subtypes [9]. Electrophysiological studies showed that baicalin mildly potentiates GABA-induced currents, and this can be abolished by co-application of the BZ site antagonist flumazenil. Thus, baicalin acts a partial BZ-site agonist, which may explain its anxiolytic activity with limited side effects. 

The anxiolytic-like effects of centrally administered baicalein were also evaluated in mice [132]. Baicalein exerts an anxiolytic-like effect already at low doses (0.02, 0.2 pmol), increasing the time spent in open arms and the head-dipping while reducing the stretched-attend postures in the elevated plus-maze. These effects are abolished by pretreatment with dehydroepiandrosterone sulfate (DHEAS) and pentylenetetrazol (PTZ) but not dl-p-chlorophenilalanine ethyl ester (PCPA) and flumazenil (FMZ), suggesting that the pharmacological activities of baicalein may dependent on GABAergic ligand sites other than BZ ones [132].

#### 3.1.2. Chronic Corticosterone-Induced Depression 

In mice models of chronic corticosterone (CORT)-induced depression, baicalin (40, 80, and 160 mg/kg) administered either orally or intragastrically produces anxyolitic- and antidepressant-like effects which are reproduced by the conventional antidepressant fluoxetine [133,134]. In fact, baicalin rescues the behavioral alterations induced by chronic CORT, namely the decreased time spent in the center and non-periphery zone in the open-field test, the increased immobility time in tail suspension test and forced swimming test, as well as the decreased time spent in open arms in the elevated plus maze test [133,134]. In chronic CORT-treated mice, baicalin restores the aberrant negative feedback of HPA axis, as assessed by dexamethasone suppression test [133]. By applying proteomics and systems biology, biological processes related to glucocorticoid receptor (GR) signaling were identified as potential molecular targets. In fact, baicalin selectively targets CORT-induced alterations in the nuclear/cytoplasmic GR distribution in the hippocampus. In detail, baicalin selectively reverses the CORT-induced decrease of GR levels in cytoplasm and the concomitant increase of GR levels in nucleus [133]. On the other hand, the CORT-induced enhancement of GR in nucleus, as well as the increased GR phosphorylation status at its crucial serine residues are similarly decreased by both baicalin and fluoxetine [133]. Baicalin, similar to fluoxetine, restores normal GR function by normalizing the levels of two GR-phosphorylating proteins, namely FK506-binding protein 51 (FKBP5) and serum- and glucocorticoid-inducible kinase 1 (SGK1) [134]. The antidepressant-like effects of baicalin are also associated with neurogenic activity. In fact, baicalin reverses the CORT-induced decrease in Ki67- and doublecortin (DCX)-positive cells in the dentate gyrus of the hippocampus [134].

#### 3.1.3. Olfactory Bulbectomy-Induced Depression 

In olfactory bulbectomy (OBX) mice models of depression, baicalin treatment (20 and 40 mg/kg) significantly reverses the abnormal levels of serum corticosterone, while normalizing behavioural alterations assessed through sucrose consumption, open field test, and forced swimming test [135]. This is associated with downregulation of the inflammatory factors IL-1β, IL-6, and TNF-α in the hippocampus and hypothalamus, which occurs through inhibition of the SIRT1-NF-kB pathway [135]. The antidepressant-like effects of baicalin (20 and 40 mg/kg) in OBX rat models are also associated with anti-oxidant and antiapoptotic activity. In fact, baicalin reverses OBX-induced alterations in the levels or activity of MDA and GSH-Px while preventing apoptotic protease-activating factor-1 (APAF-1) expression and subsequent caspase-mediated signalling cascades [136]. 

In the context of olfactory dysfunction-related depression, the effects of baicalin were evaluated in a transgenic mice model featuring hyperactivation of APPL2 (adaptor protein phosphotyrosine interacting with PH domain and leucine zipper 2). This mutation leads to depressive-like behaviour due to abnormal GR activity, impaired neurogenesis at olfactory system and loss of olfactory sensitivity [137]. Baicalin treatment blunts APPL2/GR signaling pathway and it improves neurogenesis at the subventrivular zone, olfactory bulb, and hippocampus in APPL2-Tg mice and also in a chronic CORT model. Behavioral tests revealed that baicalin similarly attenuates depressive- and anxiety-like behavior while improving olfactory functions both in APPL2-Tg mice and the chronic CORT depression model [137].

#### 3.1.4. Streptozotocin-Induced Depression 

*S. baicalensis* bioactive compounds produce antidepressant-like and cognitive-enhancing effects by targeting systemic metabolic alterations which are commonly associated with neurological and mental disorders [8]. In detail, in rat models of diabetes mellitus established via intraperitoneal injection of streptozotocin, baicalin (50, 100 and 200 mg/kg) administered daily for 7 weeks reverses depressive-like and cognitive alterations such as decreased percentage of time spent in the target quadrant, the number of times of crossing the platform in the water maze test, as well as the increase in escape latency and mean path length in the water maze test [8]. At the molecular level, baicalin counteracts streptozotocin-induced neuronal cell loss as well as the decline and increase of hippocampal acetylcholine transporter (ChAT) and acetylcholinesterase (AChE) levels, respectively. This is accompanied by increased levels of pERK, Bcl 2, and BDNF. At the same time, baicalin decreases plasma glucose levels and counteracts apoptosis by reversing the increase in phosphorylated c-Jun N-terminal kinase (pJNK), p38, caspase 3 and Bax levels [8]. 

#### 3.1.5. Chronic (unrestraint) Mild Stress-Induced Depression 

In mice models of chronic (unrestraint) mild stress (CUMS), baicalin (25, 50 and 60 mg/kg) alleviates depression-like behavior by increasing sucrose consumption and reducing immobility times in the tail suspension and forced swim tests [138,139,140]. These effects are associated with a reduction of inflammatory cytokines IL-1β, IL-6, and TNF-α levels in serum and in the hippocampus [138,139]. In mice subjected to CUMS baicalin also abrogates the increase in NMDAR/NR2B and Ca2+/calmodulin-dependent protein kinase II (CaMPK-II) as well as the decrease in pERK, while counteracting reactive oxygen species (ROS) production [138]. Baicalin administration also counteracts CUMS- and neuroinflammation (LPS)-induced depressive-like behavior in mice, and this occurs through downregulation of TLR4 via the HMBG1/NF-kb [139] and PI3K/AKT/FoxO1 pathways [140]. 

Baicalin administration exerts antidepressant-like and neuroprotective effects by counteracting oxidative stress and apoptosis in CUMS models [141]. In fact, it reduces the level of MDA, caspase-1 and IL-1β while increasing SOD in the hippocampus. These effects are mediated by inhibition of glycogen synthase kinase-3 (GSK3β)/ NF-κB / NLRP3 (Nucleotide-binding domain, leucine-rich repeat, pyrin domain containing protein 3) signaling pathway [141]. This is in line with a reduction of NLRP3 inflammasome levels which is detected in the prefrontal cortex of CUMS mice following administration of baicalin (20, 40mg/kg) [142]. 

Chronic CMS models of depression have been also employed to compare the effects of baicalin with fluoxetine [35,143]. Mice from both the baicalin and fluoxetine groups show a decrease in depression-like behavior compared with controls. Both baicalin (25, 50, 100 mg/kg) and fluoxetine (10 mg/kg) induce plastic changes by counteracting the CMS-induced decrease in the expression levels of the synaptic proteins synaptophysin (SYP), postsynaptic density protein-95 (PSD95), as well as TrkB, Rac1, cofilin and BDNF [143]. Remarkably, baicalin treatment alleviates the ultrastructural alterations occurring in the hippocampal CA3 area of the CMS group [143]. 

In another study on the CMS rat model, the effect of orally administered baicalin at the dose of 25 mg was as potent as that of fluoxetine 20 mg/kg [35]. This is associated with inhibition of monoamine oxidase A and B (MAO A/B) activity by baicalin [35]. In line with the beneficial effects of baicalin in counteracting monoamine-related alterations, these results suggest that baicalin may produce an antidepressant-like effect in vivo, at least in part, through MAO inhibition [35]. In CMS models, the antidepressant-like effects of chronic baicalin treatment (10, 20, 40 mg/kg) are also associated with anti-inflammatory activity, consisting of a reduction in the mRNA expression and activity of cyclooxygenase-2 (COX-2), as well as prostaglandin E(2) (PGE(2)) levels in the frontal cortex and hippocampus [144]. 

In CUMS mice, baicalin administration, through activation of the Akt/FOXG1 pathway, also promotes neurogenesis by increasing the number of DCX-positive cells, while fostering neuronal maturation, differentiation and survival [145]. These findings are reproduced in CUMS mice which are administered with full *Radix Scuellariae* extract (500, 1000 mg/kg) [146], which markedly reverses the shortened escape latency in morris maze test, the reduced immobility time in tail suspension test and in forced swimming test, as well as the increased sucrose consumption in sucrose preference test. These effects are associated with an enhancement of cAMP/PKA-dependent neurogenesis, as shown by the reversal of CUMS-induced reduction in BrdU, DCX and NeuN in the mice hippocampi [146]. 

Overall, these findings suggest that the antidepressant-like effects of baicalin are due to inhibition neuroinflammation and oxidative stress, potentiation of neurogenesis and neuronal differentiation, as well as amelioration of alterations associated with HPA axis hyperactivity, GABA and DA systems (Figure 2). Encouraging results are also achieved using *S. baicalensis* radix extract [146], though further experimental studies are needed to confirm its antidepressant-like action compared with baicalin alone.

### 3.2. S. baicalensis in clinical studies 

To date, clinical studies assessing specifically the antidepressant effects of *S. baicalensis* are still lacking. In the literature, there are only two available studies reporting the tolerability and the cognitive enhancing properties of *S. baicalensis* in humans. In detail, Pang et al. investigated the pharmacokinetics, safety and tolerability of baicalein after a multiple-ascending-dose protocol in thirty-three healthy Chinese volunteers. Participants were randomized to receive baicalein chewable tablets (n = 8 per dose regimen) or placebo (n = 2 per dose regimen). Dosing regimens were 200, 400, and 800 mg once daily on days 1 and 10, and twice daily on days 3–9. In the dose range of 200–800 mg, multiple-dose oral baicalein administration was safe and well tolerated [57]. 

In a human clinical trial, subjects who were orally given 300 mg of *S. baicalensis* extract (formulation UP326) for 30 days showed a marked improvement in speed and accuracy of processing complex information in computer tasks [58]. They also showed a reduced standard deviation of performance compared to baseline and the placebo group. All study compounds were well tolerated with no reports of serious or unexpected adverse effects. Thus, *S. baicalensis* may help to maintain memory, sustain speed of processing, and reduce the number or ageing-associated memory decline. Nonetheless, it cannot be argued that *S. baicalensis* has antidepressant effects in humans. This is also based on a much higher concentration of baicalin used in experimental compared with clinical studies. 

### 3.3. H. erinaceus in Experimental Studies 

Recent preclinical and clinical studies have shown that besides enhancing cognitive function and conferring neuroprotection, *H. erinaceus* also improves depression, anxiety, and sleep disturbances [39,115,147,148]. In line with this, recent studies have also been exploring the feasibility of mushrooms as potential fortified foods enriched with lithium, a well-known and gold standard mood stabilizer [4]. Co-cultivation of *H. erinaceus* with 0.25–1.0 mM lithium chloride results in a concentration-dependent uptake of lithium and its accumulation in *H. erinaceus* fruiting bodies. Such a supplementation does neither alter mushroom biomass, appearance, shape or size nor does it produce significant effects on mineral composition. As calculated, consumption of 100 g dry weight of *H. erinaceus* fruiting bodies supplemented with 1.0 mM lithium would constitute 69% of the provisional recommended dietary daily intake of lithium (1.0 mg). This suggests that *H. erinaceus* deserves to be further studied in experimental models and eventually, human studies in terms of both safety and potential synergistic activity with other herbs or conventional mood stabilizers [4]. 

Compared with saline-treated mice, dietary administration of *H. erinaceus* ethanolic fruit body extracts at 60 mg/kg once a day for 4 weeks reduces anxiety and depressive-like behaviour as assessed through elevated plus-maze, tail-suspension and forced swimming tests [149]. This is associated with increased proliferation of hippocampal progenitors and enhanced neurogenesis, which was evaluated by immunohistochemistry of proliferating cell nuclear antigen (PCNA) and Ki67 in the subgranular zone of the hippocampus, and BrdU/NeuN-positive cells in the dentate gyrus [149]. However, the present study carries an inherent limitation since healthy mice instead of depression mice models were employed as positive controls [149]. 

#### 3.3.1. Inflammation-Related Depression

The effects of *H. erinaceus* were evaluated in a mouse model of inflammation-induced depression consisting of intraperitoneal LPS administration [13]. Oral administration of the *H. erinaceus* fruit body extract amycenone (200 mg/kg) prior to a single administration of LPS (0.5 mg/kg) improves depressive-like behaviour compared with LPS-treated mice, as shown by a reduction in the immobility time measured at tail-suspension test and forced swimming tests. At the molecular level, *H. erinaceus* reduces serum levels of the pro-inflammatory marker TNF-α while increasing the anti-inflammatory cytokine interleukin-10 (IL-10). These effects are reproduced by the administration of paroxetine (30 mg/kg) suggesting that *H. erinaceus* extracts may be beneficial in inflammation-related depression [13]. 

#### 3.3.2. Restraint Stress-Induced Depression

**Figure 4:**
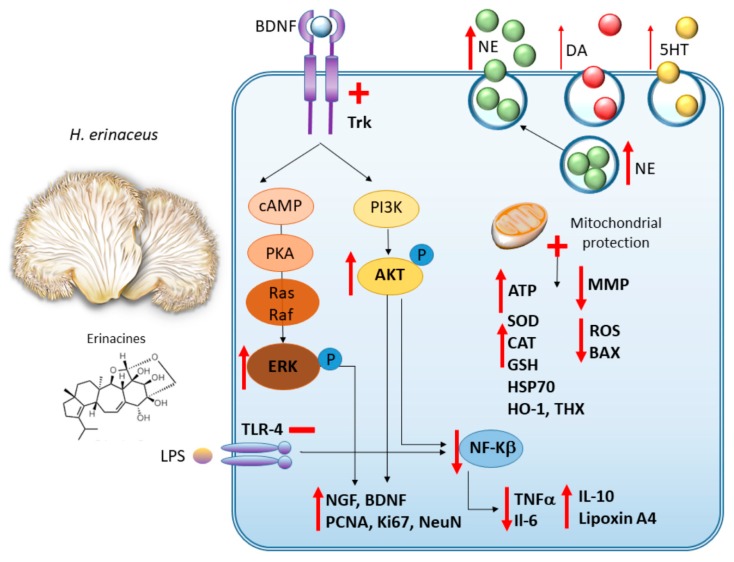
*H. erinaceus:* molecular mechanisms underlying neuroprotective and antidepressant-like effects. *H. erinaceus* and its bioactive ingredients (mostly erinacines) potentiate monoamine, and mostly norepinephrine (NE) synthesis and release. At the same time, they promote TrkB-related increase in pERK and pAKT and the subsequent synthesis of neurotrophic factors (BDNF and NGF) along with neurogenesis. *H. erinaceus* promotes anti-inflammatory effects through inhibition of TLR4/NF-kb, PI3K/AKT/Nf-κB axes. *H. erinaceus* also protects from apoptosis, mitochondrial damage and promotes anti-oxidant effects.

The findings on the anti-inflammatory and neurotrophic properties of *H. erinaceus* in relation with its antidepressant-like efficacy were recently reproduced in a mice model of repeated restraint stress (RS) [14]. Mice were orally administered *H. erinaceus* mycelium ethanolic extract at the daily dose of 100, 200 or 400 mg/kg b.w. for 4 weeks, and RS protocol was started at 2 weeks of *H. erinaceus* administration. Compared with saline-treated animals subject to RS, *H. erinaceus*, especially at the highest doses, could reverse the depressive-like behaviour caused by RS, namely the increased immobility time in the tail suspension and forced swimming tests, as well as the increased number of entries and the time spent in the open arm. This was accompanied by an increase in monoamine neurotransmitters levels along with a reduction of the pro-inflammatory factors IL-6, TNF-α and NF-κB, and the upregulation of BDNF [14]. The molecular mechanisms of action of *H. erinaceus* are summarized in Figure 4.

### 3.4. H. erinaceus in Clinical Studies 

In thirty females suffering from depression, menopause, sleep disturbances and indefinite complaints, the effects of *H. erinaceus* intake were evaluated by using the Kupperman Menopausal Index (KMI), the Center for Epidemiologic Studies Depression Scale (CES-D), the Pittsburgh Sleep Quality Index (PSQI), and the Indefinite Complaints Index (ICI) [150]. *H. erinaceus* cookies containing 0.5 g of fruit bodies powder or placebo cookies containing no powder were taken for 4 weeks. Following *H. erinaceus* treatment, each of the CES-D and the ICI score was significantly lower than that before treatment. Moreover, compared with placebo, *H. erinaceus*-receiving group showed significantly lower scores associated with insensitivity, agitation, irritation, palpitation and anxiety, suggesting that *H. erinaceus* may hold potential in reducing depression and anxiety [93]. However, no differences were observed between the two groups in the menopause and sleep-quality indexes.

A recent study carried out on 77 volunteers affected by overweight or obesity reported that a daily, 8-week oral supplementation with *H. erinaceus* (80% mycelium extract and 20% fruiting body extract), coupled with a low calorie diet regimen improves depression, anxiety, sleep, and binge eating compared with subjects undergoing low calorie diet only [91]. This is correlated with increased circulating pro-BDNF levels and pro-BDNF/BDNF ratio, despite the lack of any significant changes in BDNF circulating levels.

In an 86-year-old male patient with recurrent depressive disorder and mild cognitive impairment which developed during antidepressant therapy, Mirtazapine treatment was combined with *H. erinaceus* extract (formulation Amyloban®3399) [151]. At 6 months of *H. erinaceus* daily intake, the patient was free of depression and he showed improved cognitive function and body weight in the absence of adverse reactions, suggesting that *H. erinaceus* could be a useful antidepressant, especially in geriatric depression [151]. 

*H. erinaceus* extract (Amyloban®3399) intake for 4 weeks was also shown to counteract sleep disturbances in a pilot study on female undergraduate students [152]. This was assessed through subjective sleep-quality and well-being questionnaires GHQ-28 and PSQI (Pittsburg Sleep Quality Index) and also through the levels of salivary free- 3-methoxy-4-hydroxyphenylglycol, an index of chronic stress and depressive symptoms reflecting sympathetic nervous system activity. The latter was found to be increased after awakening, strengthening the evidence for an improvement in anxiety and sleep quality following *H. erinaceus* intake [152]. 

### 3.5. R. rosea in Experimental Studies 

#### 3.5.1. Behavioral Despair-Related Depression

The anti-depressant-like activities of *R. rosea* root’s extract have been assessed in a behavioral despair rat model of depression consisting of repeatedly forced swimming in a restricted space [36]. This leads to immobility reflecting a state of despair that is effectively reversed by antidepressant compounds in humans. Thus, the effects of *R. rosea* extracts were compared with the standard anti-depressant imipramine and with the naturally-occurring *H. perforatum* extract [36]. The herbal compounds were administered three times, once immediately after the initial exposure to the forced swimming assay, as well as 24 and 1 h prior to re-exposure. Single doses of standard anti-depressants were administered 30 min prior to stress re-exposure. *R. rosea* root extract (10, 20 and 50 mg/kg) administered either orally or intravenously increases the swimming time of rats in a dose dependent manner, and remarkably, its anti-depressant-like effects are greater compared with those produced by imipramine and *H. perforatum*. The behavior despair assay was also applied to study the effects of five single bioactive compounds isolated from *R. rosea* roots, each one administered at the dose of 0.26 mg/kg (namely rhodioloside/saliroside, tyrosol, rosavin, rosarin and rosin). Rhodioloside/saliroside exhibits higher anti-depressant effect compared with other compounds; however, the strongest effect is produced by the fixed combination of the five compounds, indicating a synergistic mechanism of action of *R. rosea* bioactive ingredients [36,99]. 

#### 3.5.2. Olfactory Bulbectomy-Induced Depression

Saliroside oral administration (20, 40 mg/kg) for 2 weeks also alleviates OBX-induced depressive-like behavior in rats, as assessed through sucrose consumption, open field test, forced swimming test and tail suspension test [19,20]. This is associated with reduced TNF-α, IL-1β, IL-6 and NF-κB levels in the hippocampus and prefrontal cortex along with a concomitant increase in BDNF expression in the hippocampus [19,20]. Besides, similarly to the antidepressant amitriptyline (10 mg/kg), saliroside markedly restores the depletion of 5-HT and NE levels in the prefrontal cortex of OBX rats [20]. Saliroside also attenuates CRF expression in hypothalamus while reducing serum corticosterone levels [19]. 

#### 3.5.3. Inflammation-Related Depression

In a mice model of LPS-induced depression, a five-day pre-treatment with saliroside (12 and 24 mg/kg), similar to fluoxetine (20 mg/kg) produces antidepressant-like behavior through anti-inflammatory, monoamine-enhancing and neurotrophic effects [153]. In detail, saliroside reduces IL-6 and TNF-α levels in the serum and NF-κB levels in the mice hippocampi. At the same time, saliroside upregulates TrkB/BDNF levels and restores 5-HT and NE levels in the prefrontal cortex of LPS-depressed mice compared with the vehicle group [153]. A recent study also evaluated the effects of both single and combined oral administration of *R. rosea* (250, 500 mg/kg) and Curcuma longa (250, 500 mg/kg) extracts in a CMS mice model of depression followed by LPS-induced inflammation [154]. The combination of *R. rosea* 500 mg/kg and Curcuma 500 mg/kg remarkably reverses depression-like behavioral changes while providing synergistic anti-inflammatory effects, namely reduced levels of TNF-α and IL-6 in CMS exposed and LPS-challenged mice [154]. 

#### 3.5.4. Chronic Mild Stress-Induced Depression

The antidepressant-like effects of *R. rosea* (1.5 g/kg/day for three weeks) in CMS rat models are also bound to stimulation of neuronal stem cells proliferation [155]. In detail, *R. rosea* improves depressive-like behavior while increasing the amount of healthy, PCNA-positive cells and preventing the occurrence of damaged, Caspase 3-positive neuronal cells [155]. 

In female CMS rat models, daily *R. rosea* administration (10, 15 and 20 mg/kg) for three weeks produces antidepressant-like behaviors which are comparable with those induced by fluoxetine (10 mg/kg). In detail, *R. rosea* reverses the CMS-induced decrease in sucrose intake, moving behaviour, minimized weight gain and dysregulation of their oestrous cycle [118]. 

In rat models of CMS-induced depression, *R. rosea* extract (1.5, 3 and 6 g/kg) administration for 3 weeks fully recovers 5-HT hippocampal levels already at the lowest dose. This is accompanied by potentiation of neural stem cell proliferation as assessed through BrdU immunohistochemistry [121]. Thus, serotonin may be crucially involved in the beneficial effects of *R. rosea* on mood disorders. These findings are reproduced in mice models showing depressive-like behavior following nicotine withdrawal [122]. In detail, in rats previously treated for 14 days with nicotine (2 mg/kg) and the selective 5-HT1A receptor antagonist WAY 100635 (1 mg/kg), *R. rosea* (5, 10, 20, and 40 mg/kg) oral administration prevents depressive-like behaviors related with nicotine withdrawal, as assessed through the abstinence scale, the immobility time on forced swimming test and marble burying test. These effects are associated with an increase in the diencephalic content of 5-HT and the expression of 5-HT1A receptor [122].

#### 3.5.5. Prepulse Inhibition-Related Depression and Psychosis

*R. rosea* effects were recently evaluated in murine models of prepulse inhibition, an established operational measure of sensorimotor gating which is impaired in a variety of mental disorders featuring psychosis [156]. In detail, *R. rosea* extract robustly reverses prepulse inhibition deficits which are induced by either the dopamine D2 receptor agonist apomorphine or the NMDA receptor antagonist dizocilpine. Thus, *R. rosea* may possess antipsychotic effects, though the underlying molecular mechanisms remain to be elucidated [156]. 

**Figure 5:**
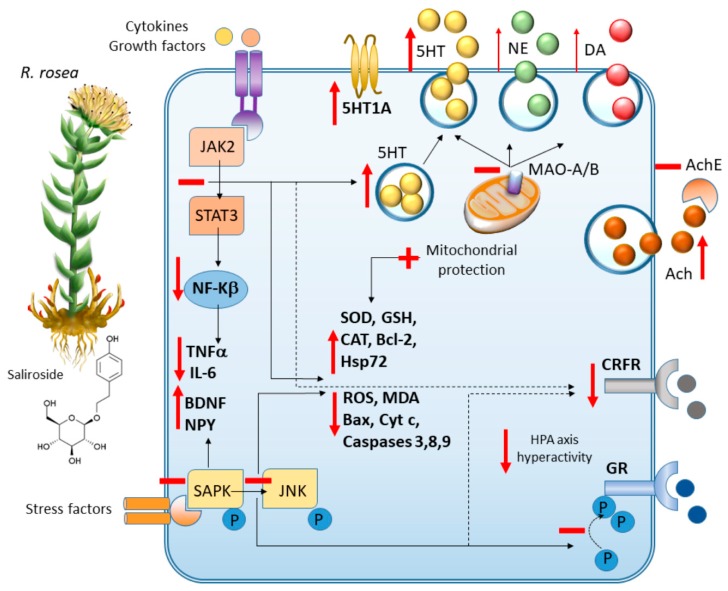
*R. rosea:* molecular mechanisms underlying neuroprotective, cognitive enhancing and anxiolytic/antidepressant-like effects. By acting as MAO A/B inhibitor, *R. rosea* and its main bioactive ingredient (saliroside) induce monoamine, and mostly 5-HT release while potentiating 5-HT synthesis and the expression of 5-HT1A receptors. *R. rosea* also potentiates Ach release while acting as an Ach esterase (AchE) inhibitor. At the same time, *R. rosea* acts as a JAK/STAT and SAPK/JNK pathway inhibitor to promote the synthesis of neurotrophic factors along with anti-inflammatory and antioxidant effects. JAK/STAT and SAPK/JNK pathway inhibition is also associated with *R. rosea*-induced downregulation of CRF receptors and inhibition of GR phosphorylation at crucial serine residues related to HPA axis activity.

In summary, the antidepressant mechanisms of *R. rosea* and saliroside might be associated with antioxidant and anti-inflammatory effects and the regulation of monoamine and cholinergic systems and HPA axis activity (Figure 5).

### 3.6. R. rosea in Clinical Studies 

In humans, *R. rosea* modulates cortical plasticity by preventing the activity-dependent reduction in neuronal synaptic efficacy [157]. A transcranial Direct Current Stimulation (DST) study evaluated the effect of a single *R. rosea* extract dose on the plastic effects induced by anodal and cathodal transcranial stimulation in twenty-eight healthy volunteers receiving 500 mg of either *R. rosea* or placebo. Acute *R. rosea* intake prevents cathodal DST-induced LTD-like plasticity, despite not affecting cortical excitability [157]. Thus, the adaptogenic, mental health-promoting effects of *R. rosea* are likely bound to plastic mechanisms of action in the brain.

#### 3.6.1. Anti-Fatigue and Anti-Stress Effects

The antiasthenic effects of *R. rosea* were evaluated by a randomized double-blind cross-over trial on a group of 56 young, healthy physicians during night duty [158]. The effects were measured as total mental performance calculated as Fatigue Index and through different perceptive and cognitive tests which were performed before and after night duty. In the *R. rosea* treatment group, significant improvements were observed in all the tests during the first two weeks’ period, and no side-effects were reported [158]. 

The effects of a standardized *R. rosea* extract (SHR-5, 50 mg) were also assessed in foreign students during a stressful examination period of 20 days [159]. The study was performed as a double-blind, randomized and placebo-controlled trial with low repeated dose regime. The physical and mental performance, which was assessed before and after the period, was based on objective as well as on subjective evaluation. Compared with placebo, significant improvements were observed in the *R. rosea* group as it concerns physical fitness and mental fatigue. The self-assessment of the general well-being was also significantly improved in the *R. rosea* group [159]. Despite these encouraging results of *R. rosea* intake, its dosage appeared suboptimal in improving mental work performance evaluated through the correction of text tests or neuro-muscular tapping test [159]. 

This issue was addressed by a subsequent clinical study which was carried out to assess the differences between the effects of two *R. rosea* doses on mental work capacity during periods of fatigue and stress [160]. This consisted of a randomized, double-blind, placebo-controlled, parallel-group clinical study on 161 cadets who were randomized into four groups, namely *R. rosea* (2x185.0 mg)-, *R. rosea* (3x185.0 mg)-, placebo-receiving and non-treatment group. The results showed a pronounced antifatigue effect along with improved work performance for both *R. rosea* dosages compared with the placebo group. No significant differences were observed between the two dosage groups. However, there was a slight trend in favour of the lower dose in the psychometric tests, which was not observed in the physiological tests [160]. Thus, *R. rosea* can effectively reduce general fatigue and anxiety under stressful conditions.

#### 3.6.2. Anxiolytic and Antidepressant Effects

A pilot study on ten adults with a diagnosis of generalized anxiety disorder (GAD) was conducted to specifically evaluate the anxiolytic efficacy of *R. rosea* [161]. Subjects receiving a daily dose of 340 mg of *R. rosea* extract for 10 weeks show significant decreases in mean Hamilton Anxiety Rating Scale (HARS) scores at endpoint, witnessing for significant improvement in GAD symptoms. Adverse events were generally mild or moderate in severity, the most common being dizziness and dry mouth [161]. 

Clinical trials of the late 1980s demonstrated that, when administered together with tricyclic anti-depressants, *R. rosea* leads to a marked reduction in the side effects of the drugs, while producing additional beneficial effects on depressive symptoms [162,163].

More recently, the antidepressant effects of *R. rosea* extract were confirmed by a phase III clinical trial which was carried out as a randomized double-blind placebo-controlled study over a period of six weeks [69]. Participants fulfilling diagnostic criteria of mild-to-moderate depression were randomized into three groups: the first (31 patients) receiving two tablets daily of *R. rosea* (SHR-5, 340 mg/day); the second (29 patients) receiving two *R. rosea* tablets twice per day (SHR-5 680 mg/day), and the third (29 patients) receiving two placebo tablets daily. The efficacy of SHR-5 extract with respect to depressive complaints was assessed on days 0 and 42 of the study period through Beck Depression Inventory (BDI) and Hamilton Rating Scale for Depression (HAMD) scores. In the groups receiving either 340 or 680 mg/day *R. rosea,* there was a significant overall improvement in depression, together with insomnia, emotional instability and somatization. No serious side-effects were reported in any of the groups, concluding that *R. rosea* exhibits anti-depressive efficacy in patients with mild to moderate depression [69].

A subsequent, randomized double-blind, 12-week, proof-of-concept trial reproduced safety and efficacy data on the antidepressant action of *R. rosea* compared with the conventional antidepressant sertraline in adult outpatients with mild to moderate depression [164,165]. Patients were randomized in three groups receiving capsules of either *R. rosea* powdered extract (SHR-5, 340 mg), sertraline (50 mg) or placebo (i.e., lactose monohydrate). The outcomes were evaluated through HRSD, BDI and Clinical Global Impression (CGI) scores. For the first two weeks all patients received one capsule daily. Subjects showing ≤ 50% reduction in HRSD score compared to baseline had the dose increased to 2 capsules daily during the following two weeks of therapy. This procedure was continued every 2 weeks for subjects with ≤ 50% reduction in HRSD score compared to baseline, up to a dose of 4 capsules daily for the last six weeks of therapy. Outcome measurements were obtained at baseline and after 2, 4, 6, 8 and 12 weeks of treatment. No statistically significant differences were observed over time for HRSD, BDI or CGI scores among treatment groups. A slightly greater decline in HRSD scores by week 12 was observed for sertraline compared with *R. rosea* and placebo. Nonetheless, clinically meaningful odds ratios of global improvement emerged by week 12 for *R. rosea* and sertraline compared with placebo; in detail, compared with placebo the odds of improvement were 1.4 and 1.9 times for *R. rosea* and sertraline, respectively. Remarkably, more subjects reported study-related adverse for sertraline (63.2%) compared with *R. rosea* (30.0%) and placebo (16.7%). While two subjects prematurely discontinued sertraline treatment, no subject prematurely discontinued *R. rosea* or placebo. In summary, *R. rosea* possesses milder antidepressant efficacy, yet, higher tolerability compared with conventional antidepressants [164,165].

The impact of an *R. rosea* extract (200 mg Vitano®) on self-reported anxiety, stress, cognition, and other mood symptoms was evaluated by a non-placebo controlled trial on eighty mildly anxious participants who were randomized into two groups, one receiving *R. rosea* twice a day and one receiving no treatments [166]. Self-report measures and cognitive tests were completed at four testing sessions over a period of 14 days. The *R. rosea*-receiving group self-reported a significant reduction in anxiety, stress, anger, confusion and depression at 14 days and a significant improvement in total mood, though no relevant differences in cognitive performance between the groups were observed. Moreover, *R. rosea* presented a favourable safety/tolerability profile [166]. 

In a recent preliminary observational study, forty-five adults suffering from mild or moderate depression were supplemented with a combination of *R. rosea* and saffron extracts (one tablet, 154 mg of *R. rosea* and 15 mg of saffron) twice a day for 6 weeks [167]. After 6 weeks (D42) of supplementation, the HARS scores decreased by 58% in nearly 85% of patients. A significant drop in both Hospital Anxiety and Depression Scale anxiety and depression scores was also observed at D42, the decrease being significant from 2 weeks of supplementation. At the end of the study, both general practitioners and patients noticed significant improvements in depression symptoms. Safety was excellent, and no serious adverse effects were recorded, suggesting that combination of *R. rosea* with other herbal compounds deserves further investigation as a potential strategy for the management of mild-moderate depression [167]. 

#### 3.6.3. Obstructive Sleep Apnea- and Menopause-Related Mood Alterations

In patients with obstructive sleep apnea (OSA), *R. rosea* was shown to relieve anxiety and depressive symptoms by inhibiting oxygen free radicals and lipid peroxidation [168]. This was assessed in ninety patients with moderate and severe OSA presenting with negative emotions diagnosed by self-rating depression scale (SDS) and self-rating anxiety scale (SAS). Compared with untreated controls, *R. rosea* intake for 3 months significantly improved SDS and SAS scores, which is associated with an increase in serum levels of SOD and a concomitant reduction of serum MDA levels [168].

As recently reviewed, *R. rosea* may also act as a potential selective estrogen receptor modulator (SERM) in the prevention and treatment of menopause-related mood disturbances, including fatigue, depression, as well as cognitive decline and memory impairment, cardiovascular disease, and osteoporosis [169]. The mechanisms of action through which *R. rosea* ameliorates menopause-related alterations include activation of intra-cellular signal transduction pathways downstream of estrogen receptors, potentiation of antioxidant defence and anti-inflammatory effects through counteracting NF-Kβ and TNF-α production [169].

A summary of both experimental and clinical studies on the antidepressant effects of *S. baicalensis*, *H. erinaceus* and *R. rosea* is provided in Table 1. 

## 4. Conclusions and Future Perspectives

The evidence here reviewed suggests that *S. baicalensis*, *H. erinaceus* and *R. rosea* deserve to be further investigated for their antidepressant/anxiolytic potential since they promote mental-health with a wide margin of safety. As common neuroprotective and antidepressant-like effects, these compounds promote synaptic plasticity and neurogenesis while counteracting oxidative stress, mitochondrial alterations, and neuro-inflammation. These three herbs also improve cognitive functions by rescuing Ach neurotransmission. These considerations are key in the light of the close association that exists among depression, cognitive alterations and neurodegeneration, being the latter often accompanied or even preceded by mood alterations [170,171]. In view of their antioxidant and mitochondrial protecting effects, these herbs could be considered as an adjunct to standard antidepressant drugs which possess side effects related to mitochondrial toxicity [172]. 

Despite common properties, each herb also produces specific, and potentially complementary effects, mostly concerning modulation of neurotransmitter systems, HPA axis, and the synthesis of neurotrophic factors. This leads to consider the chance that the combination of these different herbs may produce potentially synergistic, mood-stabilizing efficacy, which remains to be investigated. Despite a growing number of commercially available herbal formulations owning promising therapeutic potential, most of them remain untested and none have been turned into registered drugs. To date, there are many difficulties and obstacles in developing plant-derived medicines and this is due to an inadequate knowledge of their mode of action, potential adverse reactions, contraindications, and interactions with other drugs [173]. Thus, further studies are needed to deepen our understanding and knowledge on both the safety profile and fine molecular mechanisms underlying the beneficial effects of *S. baicalensis*, *H. erinaceus* and *R. rosea* in the brain. In fact, these herbal compounds may also act through yet unexplored biological pathways that are implicated in both mood and neurological disorders. For instance, some recent studies indicate that a variety of herbal compounds, including *S. baicalensis* and *R. rosea,* may produce neuroprotective effects by modulating autophagy, which is key to maintain intracellular homeostasis under oxidative stress conditions by degrading abnormal proteins or organelles, especially mitochondria [174,175,176,177]. For instance, baicalin-induced autophagy is associated with inhibition of the mitochondrial apoptotic pathway and protection against experimental traumatic brain injury [178], while saliroside-induced autophagy counteracts alpha-synuclein aggregation and toxicity [179]. The link among *S. baicalensis*, *H. erinaceus*, *R. rosea* and autophagy has not been demonstrated in depressive disorders specifically. However, such an issue deserves further investigation since autophagy alterations occur in both psychiatric and neurodegenerative disorders, and autophagy modulation is bound to the mechanisms of action of some conventional antidepressants and mood stabilizers [170,178,179,180]. 

## Figures and Tables

**Table 1:** Experimental and clinical studies centered on the antidepressant-like mechanisms and potential of *S. baicalensis*, *H. erinaceus* and *R. rosea*.

Experimental Models of Depression	*S. baicalensis*	*H. erinaceus*	*R. rosea*
Chronic corticosterone-induced depression (CORT)	Baicalin (40, 80, and 160 mg/kg) [133,134] ↑cytoplasmic GR levels ↓nuclear GR levels ↓GR phosphorylation ↑negative feedback of HPA axis [133] ↑neurogenesis (Ki67- DCX-positive cells) in the dentate gyrus of the hippocampus [134].		
Olfactory bulbectomy-induced depression (OBX)	Baicalin (20 and 40 mg/kg) [135,136,137] ↓serum corticosterone [135] ↓IL-1β, IL-6, and TNF-α in the brain via inhibition of the SIRT1-NF-kB [135] ↓oxidative stress and apoptosis [136] ↑GSH-Px and ↓MDA, APAF-1 and caspases ↑neurogenesis and olfactory function via APPL2/GR signaling pathway [137].		Saliroside (20 and 40 mg/kg) [19,20] ↓TNF-α, IL-1β, IL-6 and NF-κB in the hippocampus and prefrontal cortex ↑BDNF expression in the hippocampus [19,20] ↑5-HT and NE levels in the prefrontal cortex [20] ↓CRF expression in hypothalamus and ↓serum corticosterone levels [19].
Chronic (unrestraint) mild stress-induced depression (CUMS) and Chronic mild stress + inflammation (CUMS+LPS)	Baicalin (10, 20, 25, 40, 50, 60, and 100 mg/kg) [35,138,139,140,141,142,143,144,145] ↓monoamine oxidase A and B (MAO A/B) activity [35] ↓IL-1β, IL-6, and TNF-α in serum and in the hippocampus [138,139] ↓COX-2 and PGE(2) in the brain [144] ↓TLR4 via the HMBG1/NF-kb and PI3K/AKT/FoxO1 pathways [139,140]. ↑SOD and ↓ROS, MDA and caspase 1 [138,141] via ↓NMDAR/NR2B, ↓Ca2+/CaMPK-II and ↑pERK [139], and ↓GSK3β/ NF-κB / NLRP3 [141,142] ↓ultrastructural hippocampal alterations ↑synaptic proteins SYP PSD95, TrkB, Rac1, cofilin ↑BDNF [143] ↑neurogenesis (DCX-positive cells) ↑neuronal maturation, differentiation and survival [145]. *Radix Scuellariae* extract (500 and 1000 mg/kg) [146] ↑cAMP/PKA-dependent neurogenesis, ↑BrdU, DCX and NeuN in the mice hippocampi [146].		*R. rosea* extract (1.5 g/kg/day) [155] ↑neuronal stem cell proliferation ↑PCNA-positive cells [155] ↓Caspase 3-positive neuronal cells [155]. *R. rosea* extract (1.5, 3 and 6 g/kg) [121] ↑5-HT hippocampal levels already at the lowest dose. ↑neural stem cell proliferation ↑BrdU-positive cells [121]. *R. rosea* extract (250 and 500 mg/kg) [154] ↓TNF-α and IL-6 in CMS exposed and LPS-challenged mice [154] Potentiated effects when administered with curcuma longa (500 mg/kg) [154].
Streptozotocin (diabetes)-induced depression	Baicalin (50, 100 and 200 mg/kg) [8] ↑hippocampal acetylcholine transporter (ChAT) ↓acetylcholinesterase (AChE) ↑pERK) ↑Bcl 2 ↑BDNF ↓pJNK) ↓p-p38, ↓caspase 3 and ↓Bax [8].		
Inflammation (LPS)-induced depression	Baicalin (25, 50 and 60 mg/kg) [139,140] ↓TLR4 via the HMBG1/NF-kb and PI3K/AKT/FoxO1 pathways [139,140].	*H. erinaceus* fruit body extract amycenone (200mg/kg) [13] ↓serum TNF-α ↑IL-10 [13].	Saliroside (12 and 24 mg/kg) [153] ↓serum IL-6 and TNF-α ↓NF-κB in the brain ↑TrkB/BDNF levels ↑5-HT and NE levels in the prefrontal cortex [153].
Restraint stress-induced depression		*H. erinaceus* mycelium ethanolic extract (100, 200 and 400 mg7kg) [14] ↑monoamines levels ↓IL-6, TNF-α and NF-κB ↑BDNF [14].	
**Clinical studies**	***S. baicalensis***	***H. erinaceus***	***R.rosea***
	Baicalein chewable tablets (200, 400, and 800 mg once daily on days 1 and 10, and twice daily on days 3–9). Safe and well tolerated. Related mood effects were not analysed/reported [57]. *S. baicalensis* extract (300 mg daily for 30 days). Safe and well tolerated. Marked improvement in speed and accuracy of processing complex information in computer tasks [58].	*H. erinaceus* cookies (0.5 g of fruit bodies powder for 4 weeks). Lower scores associated with insensitivity, agitation, irritation, palpitation and anxiety in H. erinaceus-receiving women compared with placebo group [155]. *H. erinaceus* (1.650 g/day, 80% mycelium extract and 20% fruiting body extract for 8 weeks). Safe and well tolerated. Coupled with a low calorie diet improves depression, anxiety, sleep, and binge eating compared with subjects undergoing low calorie diet only [91]. Increases circulating pro-BDNF levels and pro-BDNF/BDNF ratio [91]. *H. erinaceus* extract (AmylobanⓇ) daily for 6 months combined with Mirtazapine. Combats depression, and improves cognitive function and body weight in the absence of adverse reactions [151]. *H. erinaceus* extract (AmylobanⓇ3399) intake for 4 weeks counteracts sleep disturbances in a pilot study on female undergraduate students. It increases the levels of salivary free- 3-methoxy-4-hydroxyphenylglycol, an index of chronic stress and depressive symptoms reflecting sympathetic nervous system activity [152].	*R. rosea* extract (340 mg/day for 10 weeks). Improvement of general anxiety disorder symptoms evaluated by HARS scores. Generally mild adverse effects, the most common being dizziness and dry mouth [161]. *R. rosea* extract (SHR-5, 340 or 680 mg/day for six weeks). Safe and well tolerated. Improvement of depressive symptoms, insomnia, emotional instability and somatization compared with placebo group [69]. *R. rosea* powdered extract (SHR-5 capsule, 340 mg, one capsule/day for the first week, two capsules/day for the following two weeks, up to up to 4 capsules/day for the last six weeks). Improves depressive symptoms compared with placebo and produces antidepressant effects which are comparable with sertraline (50 mg). Fewer adverse effects were reported for R. rosea compared with sertraline [164,165]. *R. rosea* extract (Vitano®, 200 mg twice a day for 14 days). Safe and well tolerated. Reduces self-reported anxiety, stress, anger, confusion and depression, and overall improvement in mood [166] *R. rosea* extract (one tablet, 154mg, combined with saffron tablet 15 mg, twice a day for 6 weeks). Excellent safety, no serious adverse effects. Improvements in HARS scores and depression symptoms reported by both general practitioners and patients [167].

## Abbreviations

6-OHDA	6-hydroxydopamine
AChE	acetylcholinesterase
AD	Alzheimer’s disease
APPL2	adaptor protein phosphotyrosine interacting with PH domain and leucine zipper 2
BBB	blood-brain barrier
BDI	Beck Depression Inventory
BDNF	brain-derived neurotrophic factor
BZ	benzodiazepine
CAT	catalase
CES-D	Center for Epidemiologic Studies Depression Scale
CGI	Clinical Global Impression
ChAT	acetylcholine transporter
CORT	corticosterone
COX-2	cyclooxygenase-2
CRF	corticotrophin-releasing factor
CRH	corticotropin-releasing hormone
CUMS	chronic (unpredictable) mild stress
D1Rs	DA D1 receptors
DA	dopamine
DAT	DA transporter
DCX	doublecortin
DPPH	1-diphenyl-2-picrylhydrazyl
DST	Direct Current Transcranial Stimulation
GABA	Gamma-Aminobutyric acid
GABA_A_R	GABA A subtype receptor
GAD	generalized anxiety disorder
GR	glucocorticoid receptor
GSH	glutathione
GSH-Px	glutathione peroxidase
GSK3β	glycogen synthase kinase-3
H2O2	hydrogen peroxide
HAMD	Hamilton Rating Scale for Depression
HARS	Hamilton Anxiety Rating Scale
HD	Huntington’s disease
HMBG1	High Mobility Group Box 1
HPA	hypothalamic-pituitary-adrenal axis
HSP70	heat shock proteins 70
ICI	Indefinite Complaints Index
IL-1β	interleukin 1 beta
KMI	Kupperman Menopausal Index
LC	Locus Coeruleus
LPS	Lipopolysaccharide
MAO A/B	monoamine oxidase A/B
MDA	malondialdehyde
METH	methamphetamine
MMP	mitochondrial membrane potential
MPTP	1-methyl-4-phenyl-1,2,3,6-tetrahydropyridine
NE	norepinephrine
NF-κB	nuclear factor kappa-light-chain-enhancer of activated B cells
NGF	nerve growth factor
NLRP3	nucleotide-binding domain, leucine-rich repeat, pyrin domain containing protein 3
NMDAR/NR2B	N-methyl D-aspartate receptor subtype 2B
NPY	neuropeptide Y
OBX	olfactory bulbectomy
OSA	obstructive sleep apnea
PCNA	proliferating cell nuclear antigen
PD	Parkinson’s disease
pERK	phosphorylated Extracellular signal-regulated kinase
PGE(2)	prostaglandin E(2)
pJNK	phosphorylated c-Jun N-terminal kinases
PSD95	postsynaptic density protein-95
PSQI	Pittsburgh Sleep Quality Index
ROS	reactive oxygen species
RS	repeated restraint stress
SAS	self-rating anxiety scale
SDS	self-rating depression scale
SERM	selective oestrogen receptor modulator
SOD	superoxide dismutase
SYP	synaptophysin
TLR4	toll-like receptor 4
TNF-α	tumor necrosis factor alpha
TrkB	tyrosine kinase receptor B
